# Out-of-Plane Coordinated Porphyrin Nanotubes with Enhanced Singlet Oxygen Generation Efficiency

**DOI:** 10.1038/srep31339

**Published:** 2016-08-16

**Authors:** Qiang Zhao, Yao Wang, Yanshuang Xu, Yun Yan, Jianbin Huang

**Affiliations:** 1 Beijing National Laboratory for Molecular Sciences (BNLMS), State Key Laboratory for Structural Chemistry of Unstable and Stable Species, College of Chemistry and Molecular Engineering, Peking University, Beijing 100871, People’s Republic of China; 2 Beijing National Laboratory for Molecular Sciences (BNLMS), Key Laboratory of Polymer Chemistry and Physics of the Ministry of Education, College of Chemistry and Molecular Engineering, Peking University, Beijing 100871, People’s Republic of China

## Abstract

A supramolecular porphyrin nanotube displaying J-aggregation feature was constructed by out-of-plane coordinated bismuth-porphyrin. Significantly, compared to traditional J-aggregated porphyrin suffering from fluorescence and singlet oxygen quenching, the nanotube exhibits excellent bio-imaging ability and enhanced production efficiency of singlet oxygen. The out-of-plane structure of bismuth to porphyrin makes the aggregation an appropriate material for theranostics. Furthermore, it is also a potential radio-therapeutic drug owing to the presence of radio-active bismuth. Thus, the self-assembly of out-of-plane coordinated porphyrin can be a facile approach toward effective therapy of tumors and other diseases.

Photodynamic therapy (PDT) is a clinical method to eradicate early-stage cancer and to reduce the size of tumor in the end-stage cancer^1^,^2^,^3^, which includes the combination of a photosensitizer, light and molecular oxygen (^3^O_2_)^4^,^5^. When exposed to the appropriate light, the photosensitizers can produce cytotoxic oxygen species leading to a selective and effective damage of diseased cells and tissues^6^,^7^. Hence, the photosensitizers with high singlet oxygen (^1^O_2_) quantum yield are desirable^8^. Porphyrins, as one of promising photosensitizers, have received much attention in PDT for their intense absorption and excellent singlet oxygen generation quantum yield^9^. Upon photo irradiation in UV-visible region, porphyrins transfer energy to triplet oxygen (^3^O_2_) and generate cytotoxic single oxygen (^1^O_2_) which causes cellular and tissue damage ultimately^10^,^11^.

However, the large π-conjugate of planar porphyrin often undergoes obvious aggregation which causes severe quenching of the excited state thus leads to the decrease of ^1^O_2_ generation^12^,^13^. It greatly limits the applications in biomedical field, especially in PDT application. To address this important issue, some space-demanding porphyrins have been introduced to segregate porphyrin units and suppress the quenching effect, such as co-assemble porphyrins with bulky molecules^14^, load porphyrins to other nanostructures^15^,^16^,^17^, and introduce steric moieties *via* organic synthesis^18^,^19^,^20^, *etc*. In these processes, the strategies often involve complicated chemical synthesis, which is time-consuming and rises the risk of cytotoxicity due to the use of organic solvents. Therefore, pursuing a non-covalent method to make functional porphyrin nanostructures still remains challenging.

Metal porphyrins are known to facilitate the energy transfer *via* intersystem crossing^21^,^22^, which is of key importance in the generation of singlet oxygen for PDT. Some metals coordinated with porphyrin and formed an out-of-plane structure that break the planar geometry and orderly arrangement of porphyrin^23^,^24^,^25^. Unfortunately, out-of-plane coordination can hardly drive well-defined porphyrin nanostructures so far. If the out-of-plane coordinated porphyrin can be employed properly, porphyrin nanostructures could be generated without close π-π stacking. It is of significant importance for suppressing the self-quenching in porphyrin aggregations and enhancing the efficiency of singlet oxygen generation.

Herein we report a rare case of J-aggregated porphyrin nanotubes with enhanced singlet oxygen generation efficiency. Tetrasodiummeso-tetra(sulfonatophenyl)porphine (TPPS_4_) was employed to react with Bi(NO_3_)_3_ to generate TPPS_4_-Bi nanotube. TPPS_4_ is widely used as model porphyrins to study their self-assembling behaviors^26^,^27^. In this work, the TPPS_4_ was found to self-assemble into nanotubes with Bi^3+^ by out-of-plane coordination. The coordination of Bi^3+^ makes a staggered arrangement of porphyrins so that it could reduce the overlap of porphyrin planar and suppress the self-quenching in aggregation (Fig. 1). Surprisingly, the nanotube exhibited an enhanced production of singlet oxygen similar to the monomeric porphyrins and an excellent fluorescence imaging ability in cells. Moreover, considering that Bi^3+^ also has wide applications in radio-therapy for the treatment of tumors and gastrointestinal disorders^28^,^29^, the TPPS_4_-Bi nanotube is expected to multiple functions as theranostics. The self-assembly of porphyrin which could suppress self-quenching without other co-assembling molecules or covalent modification is still challenging. Therefore, the present work opens a new vista to construct more porphyrin photosensitizers for the therapy of tumors and other diseases.

## Results

The TPPS_4_-Bi nanotube is fabricated in aqueous solution at pH 3.6, which is a condition that prohibits the hydrolysis of Bi^3+^. In such acidic solution, TPPS_4_ only could self-assemble into flexible and fiber-like J-aggregates which suspended in the solution (Supplementary Fig. S1)^30^,^31^,^32^,^33^. However, upon addition of Bi(NO_3_)_3_, precipitates were formed. TEM and SEM images demonstrate that the precipitates are composed of nanotubes with lengths and widths around 500 ± 100 nm and 50 ± 5 nm, respectively (Fig. 2). The wall-thickness of these nanotubes is only about 10–20 nm. EDX analysis reveals the presence of Na and Bi, suggesting these nanotubes are formed with TPPS_4_ and Bi(NO_3_)_3_ (Supplementary Fig. S2).

Supramolecular structure of TPPS_4_-Bi nanotubes was investigated using UV-Vis, fluorescence and circular dichroism (CD) spectroscopy. The TPPS_4_ in aqueous solution displays two UV-Vis absorption bands at 434 and 645 nm. Upon addition of equimolar Bi^3+^, these two bands gradually red shift to 491 and 707 nm, respectively, suggesting an increased extent of J-aggregation (Fig. 3a)^30^,^31^,^32^,^33^. Meanwhile, the fluorescence emission blue shifts from 680 nm to 516 nm, confirming that Bi^3+^ has induced further J-aggregation of TPPS_4_ (Supplementary Fig. S3).

**Figure 1:**
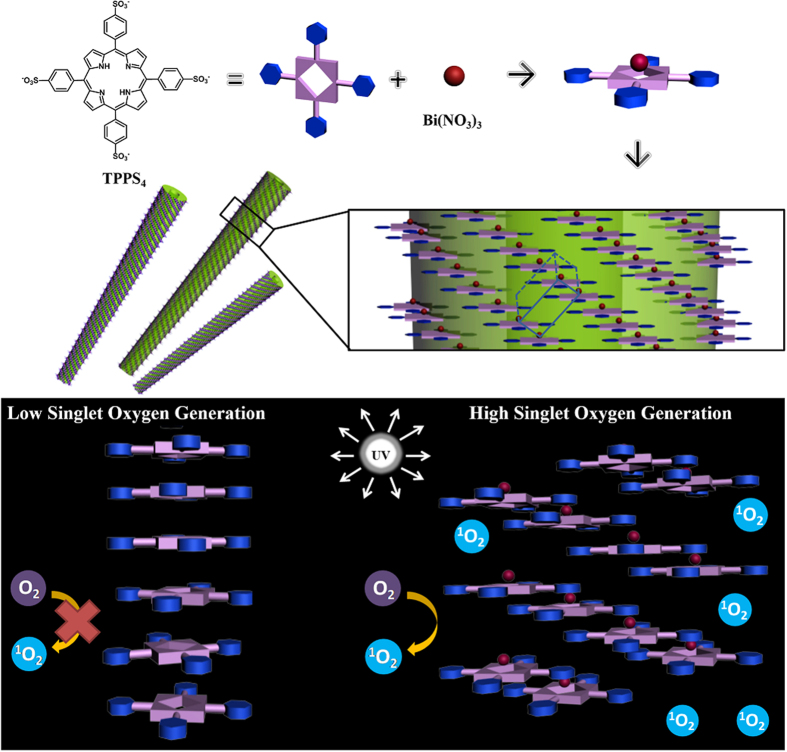
Chemical structures of the TPPS_4_ and the mechanism for the enhanced singlet oxygen efficiency of TPPS_4_-Bi nanotubes compared with that of TPPS_4_ aggregation.

**Figure 2:**
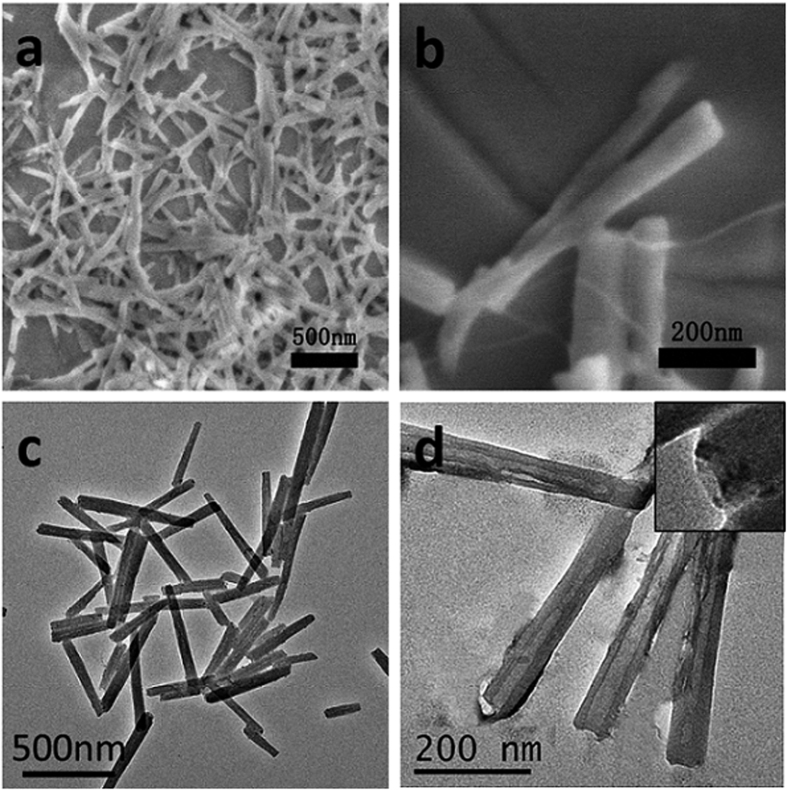
The SEM (**a,b**) and TEM (**c,d**) images of TPPS_4_-Bi nanotubes. [TPPS_4_] = 0.1 mM, [Bi^3+^] = 0.2 mM, pH 3.6.

**Figure 3:**
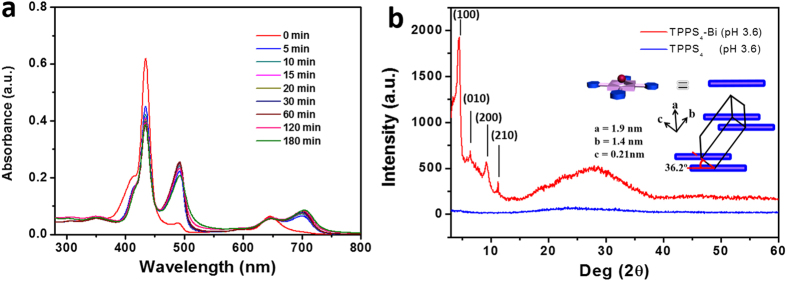
(**a**) Time dependent UV-Vis absorption of TPPS_4_-Bi aqueous solution. [TPPS_4_] = 0.025 mM, [Bi^3+^] = 0.05 mM (pH 3.6). (**b**) XRD measurements for the TPPS_4_-Bi nanotubes and TPPS_4_ aggregates in acid aqueous solution (pH 3.6). The inset in (**b**) shows the arrangement of the TPPS_4_ molecules in one cubic cell. The dislocation angle between the neighboring top and down TPPS_4_ plane was 36.2°.

Figure 3b shows the X-ray diffraction (XRD) data for the TPPS_4_-Bi nanotubes. Compared with J-aggregation of TPPS_4_ only^30^,^31^,^32^,^33^, sharp brag diffractions occur in TPPS_4_-Bi nanotubes, suggesting Bi^3+^ has induced orthorhombic TPPS_4_ crystal cells with a dimension of a = 1.9 nm, b = 1.4 nm, and c = 0.21 nm. It is noteworthy that Bi^3+^ coordinates to TPPS_4_ *via* out-of-plane coordination due to its large size with radius about 0.1 nm^23^. The distance between two neighboring TPPS_4_ face is only 0.21 nm, much smaller than that of the 0.3 nm for the usual J-aggregated TPPS_4_^25^. The electrostatic interactions between the Bi^3+^ of one TPPS_4_ and the SO_3_^−^ of another one decrease distance between two TPPS_4_ and result in the formation of the J-aggregated TPPS_4_-Bi nanotubes. Based on the crystal cell parameters, the coordinated Bi^3+^ locates in between two dislocated TPPS_4_ planes and makes porphyrins arrange in a dislocation angle at about 36.2°, which is much smaller than the critical dislocation angle of 54.7° featuring J-aggregation^34^. As a result, the dislocation of porphyrin induces the stair-case type self-assembly of TPPS_4_ as illustrated in Fig. 1 and greatly reduces the extent of overlapping of porphyrin rings. In this way, the π-π stacking overlap was significantly reduced. Since the radius of Bi^3+^ is only around 0.1 nm which did not trigger extra steric hindrance for the arrangement of TPPS_4_, no induced chiral signal was observed upon formation of the nanotube (Supplementary Fig. S4). It is noticed that NO_3_^−^ could exist in nanostructure by coordination interaction between Bi and NO_3_^−^35. In addition, the TPPS_4_-Bi nanotubes can be viewed as a nanostructure with 100% loading of porphyrins because no additional co-assembling molecules were added into the system.

The suppression of self-quenching in TPPS_4_-Bi was confirmed with time-resolved fluorescence studies (Supplementary Fig. S5 and Table 1). In neutral solution, 84% TPPS_4_ exhibits a lifetime of 11.75 ns corresponding to the monomeric TPPS_4_^36^. Before addition of Bi^3+^, 94% porphyrin in J-aggregated TPPS_4_ at pH 3.6 exhibits a short lifetime of 3.8 ns, which indicates that the π-π stacking overlap of TPPS_4_ in J-aggregation have resulted in self-quenching of porphyrin. However, the lifetime is remarkably enhanced in J-aggregated TPPS_4_-Bi system at pH 3.6, 65% TPPS_4_ has the unprecedented long lifetime of about 11.10 ns, similar to that of monomeric TPPS_4_ (Table 1). In addition, the long lifetime luminescence of TPPS_4_-Bi corresponds with triplet stated that the emission at 600 nm could be quenched by oxygen^37^, as shown in Supplementary Fig. S6. It is suggested that TPPS_4_-Bi system possesses the potential in generation of singlet oxygen. The tubular structures and presence of heavy atom both promoted the intra-system crossing (ISC) which leads to the enhancement of photophysical properties.

**Figure 4:**
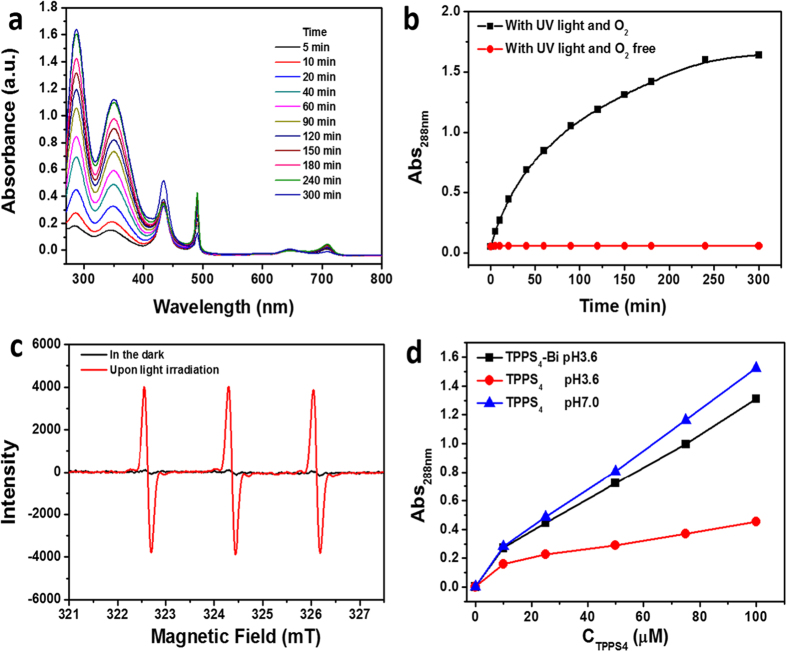
Time depending (**a**) UV-Vis spectra of the TPPS_4_-Bi nanotubes in the presence of KI and (**b**) the comparison of TPPS_4_-Bi nanotubes in the presence of UV light and O_2_ (□) and O_2_ free (○), respectively. The absorption was at 288 nm. (**c**) EPR spectra of the TPPS_4_-Bi nanotubes solution in which trace TEMP acts to capture the ^1^O_2_ generated in the dark or upon light irradiation for 10 min. (**d**) Compared singlet oxygen generation tested by UV-Vis spectra at λ = 288 nm with increasing concentrations at TPPS_4_ (pH 7.0), TPPS_4_ (pH 3.6) and TPPS_4_-Bi (pH 3.6) systems. [TPPS_4_] = 0.025 mM, [Bi^3+^] = 0.05 mM, C_KI_ = 1 M.

The long lifetimes of porphyrin in TPPS_4_-Bi system allow the nanotubes display excellent ability of singlet oxygen generation which was verified by iodide method and electron paramagnetic resonance (EPR) spectroscopy. The principle of this method is that the amount of I_3_^−^ produced by oxidation of I^−^ with ^1^O_2_ is proportional to the concentration of ^1^O_2_ under continuous irradiation^38^. The light induced oxidation of iodide is chosen as model reactions for catalysis as Scheme S1. Figure 4a,b show that upon irradiation with UV light, the characteristic absorption of I_3_^−^ at λ = 350 nm and λ = 288 nm occurred, indicating the generation of ^1^O_2_ depending on time increasing. EPR spectroscopy was also employed to monitor the ^1^O_2_ generation ability of TPPS_4_-Bi upon light irradiation. Trace amounts of 2,2,6,6-tetramethylpiperidine (TEMP), which is a diamagnetic and water-soluble molecule, was applied to capture ^1^O_2_ by yielding a paramagnetic product TEMPO^39^,^40^. The unpaired electron located on the NO group of TEMPO can lead to the hyperfine splitting of the EPR signal into three narrow lines, arising from the interaction between the unpaired electronic spin and the nitrogen nucleus. When the oxygen-saturated solution of TPPS_4_-Bi was irradiated in the presence of TEMP for 10 min, the EPR signal increased as shown in Fig. 4c. The result of EPR test was in accordance with iodide method which confirmed the generation of singlet oxygen in TPPS_4_-Bi system.

Moreover, compared with self-quenching J-aggregated TPPS_4_ at pH 3.6, the TPPS_4_-Bi nanotubes display a significantly enhanced singlet oxygen production (Fig. 4d). It is noteworthy that ability of singlet generation for TPPS_4_-Bi nanotubes is comparable to that of monomeric porphyrins, which is probably benefited from the reduced π-π stacking by staggered porphyrin arrangement. Moreover, the nanotubes remain stable when dispersed in neutral water and the singlet oxygen generation ability was not apparently affected by changing pH (Supplementary Fig. S7). When the TPPS_4_-Bi nanotubes were dispersed and irradiated by light in neutral solution or in oxygen free solution, there is no obvious generation of ^1^O_2_ (Figs 4b and S6).

**Figure 5:**
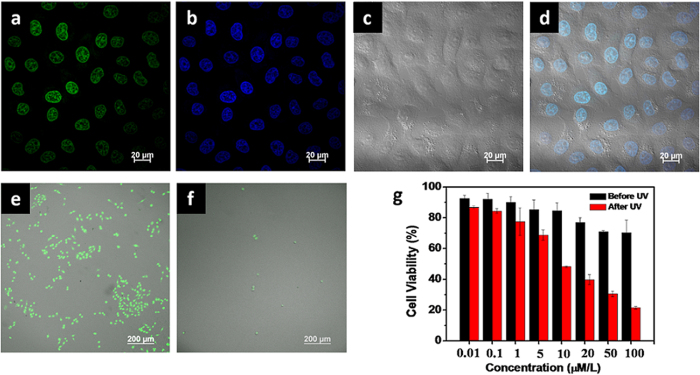
Confocal images of HeLa cells labeled with Hoechst 33258 after incubating with TPPS_4_-Bi nanotubes. The fluorescent fields after (**a**) treated with 10 μM mL^−1^ TPPS_4_-Bi nanotubes and (**b**) Hoechst 33258 stained. (**c**) The optical field and (**d**) the overlap images of bright and fluorescence field in HeLa cells. The overlap images of bright field and fluorescence field (**e**) before and (**f**) after light irradiation in HeLa cells which were treated with 10 μM mL^−1^ TPPS_4_-Bi nanotubes. (**g**) SRB test for the cytotoxicity of TPPS_4_-Bi nanotubes to HeLa cells after 48 hours incubation in various concentrations before and after UV irradiation. Data were presented as the mean ± standard deviation (N = 3).

The excellent singlet oxygen generation ability of the TPPS_4_-Bi nanotube in neutral solution indicates a potential application in cells. We incubated the TPPS_4_-Bi nanotubes with HeLa cell for 24 h. The cells are lighted up by the nanotube (Fig. 5a), and the strength of the green fluorescence is comparable to that of the commercial lysosome imaging dye Hoechst 33258 (Fig. 5b). The green and the blue window overlapped perfectly and resulted in the cyan fluorescence in the cell nucleus (Fig. 5c,d). Since the fluorescence of porphyrins is usually obtained as they exist in the isolated states, the present results indicate that the out-of-plane coordination have retained the monomeric porphyrin, which is in agreement with the detection of long lifetimes in time resolved fluorescence measurements.

The sufficient fluorescence of TPPS_4_-Bi in HeLa cells manifests the possibilities of using this nanotube as theranostics, far beyond function solely as the PDT drug. Therefore, the PDT effect of the nanotube was tracked with its own fluorescence, rather than under the help of commercial dyes. Figure 5e,f are the images of the HeLa cells treated with 10 μM TPPS_4_-Bi nanotubes before and after irradiation. Before irradiation, large amount of living cells are lighted up. In contrast, only several cells are observed after 10 min’s irradiation followed by washing out the dead cells with water. A qualitative SRB analysis in Fig. 5g shows that half of the HeLa cells have been killed to death under the same condition. However, controlled experiments suggest that no cell death can be observed without irradiation. Meanwhile, blank test manifests that irradiation of untreated cells would not lead to considerable cell death, too (Supplementary Fig. S8), showing that the TPPS_4_-Bi nanotubes display excellent phototoxicity.

In summary, we have prepared TPPS_4_-Bi nanotubes through the facile out-of-plane coordination assisted self-assembly. The Bi^3+^ locates between two porphyrins at a dislocation angle which induces staggered arrangement of porphyrins and prevents π-π stacking overlap. It allows the TPPS_4_-Bi J-aggregation to suppress the self-quenching effect and display an enhanced singlet oxygen generation compared with traditional TPPS_4_ J-aggregation. Since the component Bi^3+^ is also a radio-therapy agent for tumors and other diseases, and there are judicious choices of functionally large metal ions and porphyrins, we expect that the out-of-plane coordination approach inspires much exciting design of porphyrin aggregations for multifunctional theranostics.

## Methods

### Materials

The tetrasodiummeso-tetra(sulfonatophenyl)porphine (TPPS_4,_ >97.0%) was purchased from Alfa Aesar. Bi(NO_3_)_3_·5H_2_O, Zn(NO_3_)_3_, Pb(NO_3_)_3_ and TiCl_4_ were purchased from Beijing Chemical Reagents (Beijing, China, >99.0%). All the aqueous solutions were prepared using Milli-Q water (Millipore, 18 MΩ/cm resistivity).

### Preparation of TPPS_4_-Bi Nanotubes

The porphyrin nanostructure were fabricated by mixing aqueous solutions of TPPS_4_ and Bi(NO_3_)_3_ at pH 3.6. Typically, 0.1 mL of H_4_TPPS_4_^2−^ solution (1 mM) was mixed with 0.2 mL of Bi(NO_3_)_3_ solution (1 mM) in 0.7 mL water, and the mixture as left undisturbed in the dark at 25 °C for 24 h. Over time, it provides a high yield of nanostructure.

### Scanning Electron Microscopy (SEM)

A drop of TPPS_4_-Bi suspending solution was placed on clean silicon sheets and dried freely under ambient conditions. The samples were observed by a SEM, Hitachi S4800, 5 kV.

### Transmission Electron Micrograph (TEM)

Samples were observed by a Jeol JEM 100 CX, 80 kV and JEM-2100, 200 kV, together with energy-dispersive spectroscopy (EDS) measurement. Drops of samples were put onto 230 mesh copper grids coated with formvar film. Excess water was removed by filter paper and the samples were allowed to dry in ambient air at RT, before TEM observation. It is noticed that all samples were not stained except for the one which was made of TPPS_4_ only.

### Spectra Measurements

UV-Vis absorbance measurements were carried out on the Beijing Purkinje General Instrument Co. Ltd. TU-1810. The generation of singlet oxygen measured by UV-Vis absorption was carried out at 288 nm. The fluorescent measurements were tested by a fluorescent spectrophotometer, Hitachi F7000. The lifetime measurements of all samples were tested by a lifetime and steady state spectrometer (Edinburgh Instruments Ltd. FLS920). The circular dichroism (CD) spectrum was tested by a circular dichroism chiroptical spectrometer (Jasco Co.). All the spectra measurements were conducted at RT.

The light induced oxidation of iodide is chosen as model reaction for catalysis as previous reports. The reaction was shown in Scheme S1. The solution was deoxygen by nitrogen and kept in dark for 24 h. Before test, the solution was flowed by oxygen and added with KI. Then light was applied to induce the generation of ^1^O_2_. The ^1^O_2_ generation efficiency was tested by UV-Vis measurement at 288 nm absorption.

### X-Ray Diffraction (XRD)

For XRD measurements, several drops of suspension of TPPS_4_ and TPPS_4_-Bi were dropped on a clean glass slide respectively, followed by drying in the air. All samples were tested by an instrument (Rigaku Dmax-2000, Ni-filtered Cu Kα radiation) under ambient conditions at RT.

### Singlet Oxygen Measurement

The EPR spectroscopy was used to monitor the generation of singlet oxygen in aqueous solutions. Singlet oxygen was detected as TEMP-^1^O_2_ adduct (TEMPO) using TEMP as a singlet oxygen trap. The EPR experiments were performed at room temperature on a JEOL JES-FA200 apparatus. The solution was saturated with oxygen, followed by addition of trace amount of TEMP and then irradiated by a xenon lamp with a sharp-cut filter (the cut off wavelength is 450 nm). The TEMPOL signal was analyzed by EPR.

### Confocal Laser Scan Microscopy (CLSM)

The samples were dropped on a clean glass slide and covered by a clean cover slip. A TCS-sp inverted confocal laser scanning microscope (Leica, Germany) was used to conduct experiments in florescence and differential interference contrast (DIC) modes.

### Cell Culture and Cytotoxicity Assay *in Vitro*

In this study, HeLa cell lines were used. The HeLa cells (Institute of MateriaMedica, Chinese Academy of Medical Sciences and Peking Union Medical College, Beijing, China) were routinely grown in DMEM medium supplemented by 10% heated-inactivated fetal bovine serum (FBS), 100 U/mL penicillin and 100 mg/mL streptomycin. Cells were maintained at 37 °C with 5% CO_2_. For cytotoxicity tests, HeLa cells were seeded into 96-well culture plates at a density of 5 × 10^3^ cells/well and grown for 24 h. Then TPPS_4_-Bi nanotubes were added into 96-well culture plates. The concentration of TPPS_4_-Bi nanotubes were in the range of 1 × 10^−3^ − 50 μM mL^−1^. Then after 48 h incubation, the cell viability was measured by a microplate reader at 540 nm with the SRB staining assay. The following formula was used: Survival% = (A_540nm_ for the treated cells/A_540nm_ for the control cells) × 100%, where the A_540nm_ was the absorbance value. Each assay was repeated for 5 times.

### Confocal Microscopic Imaging of Cells Using TPPS_4_-Bi Nanotubes

The HeLa cells were seeded into 96-well culture plates at a density of 5 × 10^3^ cells/well and grown for 24 h. The cells were taken with 4 μg mL^−1^ TPPS_4_-Bi nanotubes for 4 h at 37 °C. Afterward, the cells were washed three times with PBS to remove the non-internalized nanotubes and incubated for another 24 h in a 24-well plate. Then the cells were fixed with 4% paraformaldehyde for 10 min and stained with 10 μg mL^−1^ Hoechst 33258 at room temperature. Cell images were taken with a confocal laser scanning microscope with the excitation wavelengths of 405 nm and 488 nm.

## Additional Information

**How to cite this article**: Zhao, Q. *et al*. Out-of-Plane Coordinated Porphyrin Nanotubes with Enhanced Singlet Oxygen Generation Efficiency. *Sci. Rep.* **6**, 31339; doi: 10.1038/srep31339 (2016).

## Supplementary Material

Supplementary Information

## Figures and Tables

**Table 1:** The longest fluorescence lifetime (τ) of TPPS_4_ under different conditions.

	pH	τ/ns	Percentage of molecules with each lifetime (%)	Fitting quality χ^2^
TPPS_4_	6.8	11.75	84.03	0.9444
TPPS_4_	3.6	3.783	94.13	1.062
TPPS_4_-Bi	3.6	11.10	65.34	1.295

The fraction of molecules with long lifetimes was given as hundred percent (%). The fitting quality was expressed by the parameter χ^2^.
