# Supplementary material for: Out-of-Plane Coordinated Porphyrin Nanotubes with Enhanced Singlet Oxygen Generation Efficiency
Source: Sci Rep. 2016 Aug 16;6:31339. doi: 10.1038/srep31339 (PMC4985641; doi:10.1038/srep31339)

**Supporting Information**

**Out-of-Plane Coordinated Porphyrin Nanotubes with Enhanced Singlet Oxygen Generation Efficiency**

*Qiang Zhao†, Yao Wang**‡, Yanshuang Xu‡, Yun Yan†*, Jianbin Huang†**

†Beijing National Laboratory for Molecular Sciences (BNLMS), State Key Laboratory for Structural Chemistry of Unstable and Stable Species, College of Chemistry and Molecular Engineering, Peking University, Beijing 100871, People’s Republic of China;

‡Beijing National Laboratory for Molecular Sciences (BNLMS), Key Laboratory of Polymer Chemistry and Physics of the Ministry of Education, College of Chemistry and Molecular Engineering, Peking University, Beijing 100871, People’s Republic of China.


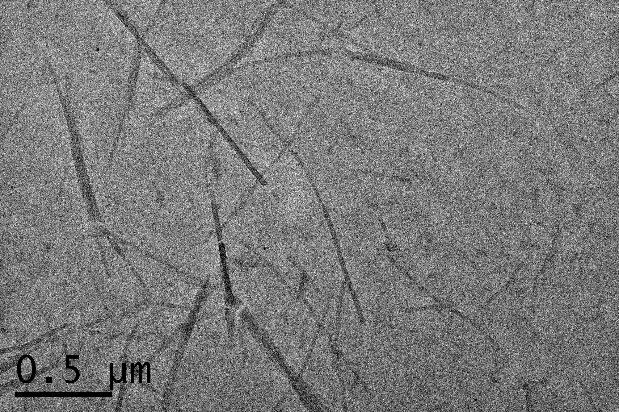

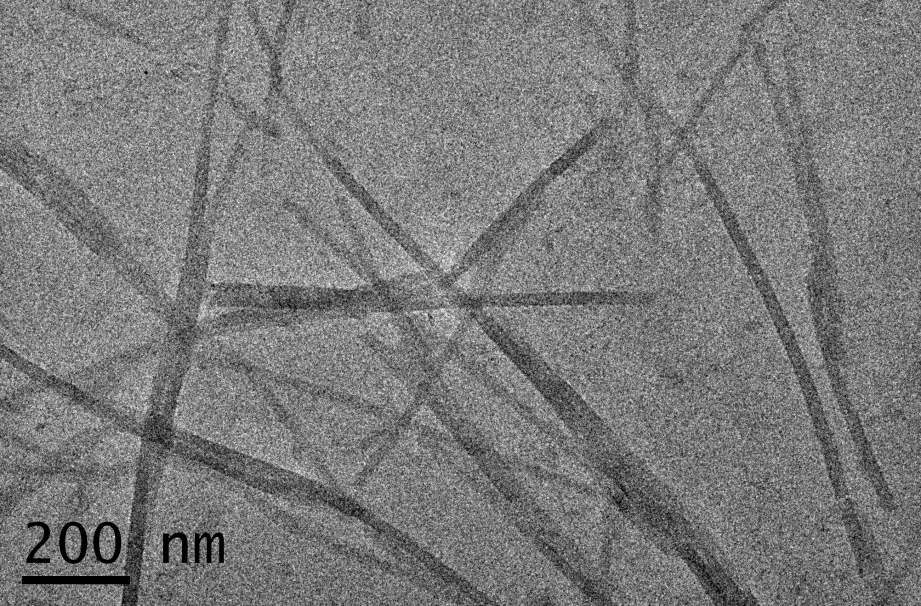


**Figure S1**. (a)(b)The TEM image of TPPS_4_ in acid solution (pH 3.6).


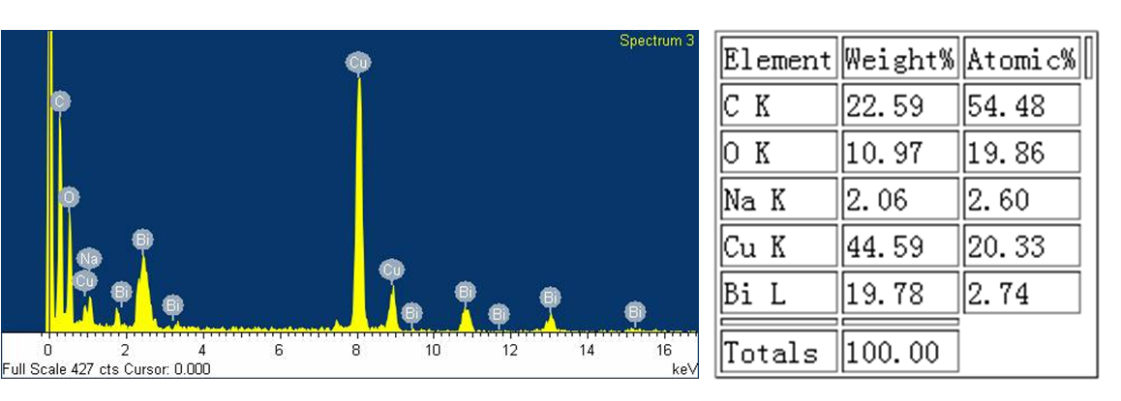


**Figure S2**. The EDX image of TPPS_4_-Bi nanotubes and analysis of each element.


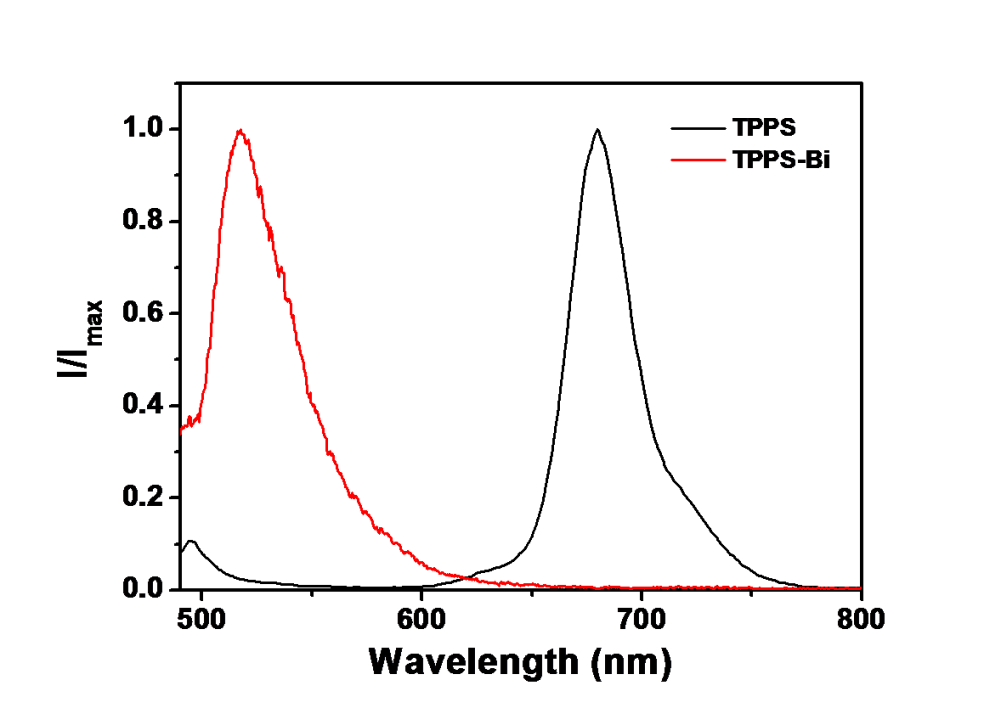


**Figure S3.** The fluorescence of TPPS_4_ and TPPS_4_-Bi aqueous solution with excitation wavelength at 450 nm and 415 nm. [TPPS_4_] = 0.025 mM, [Bi^3+^] = 0.05 mM, pH 3.6. Excitation, 370 nm.


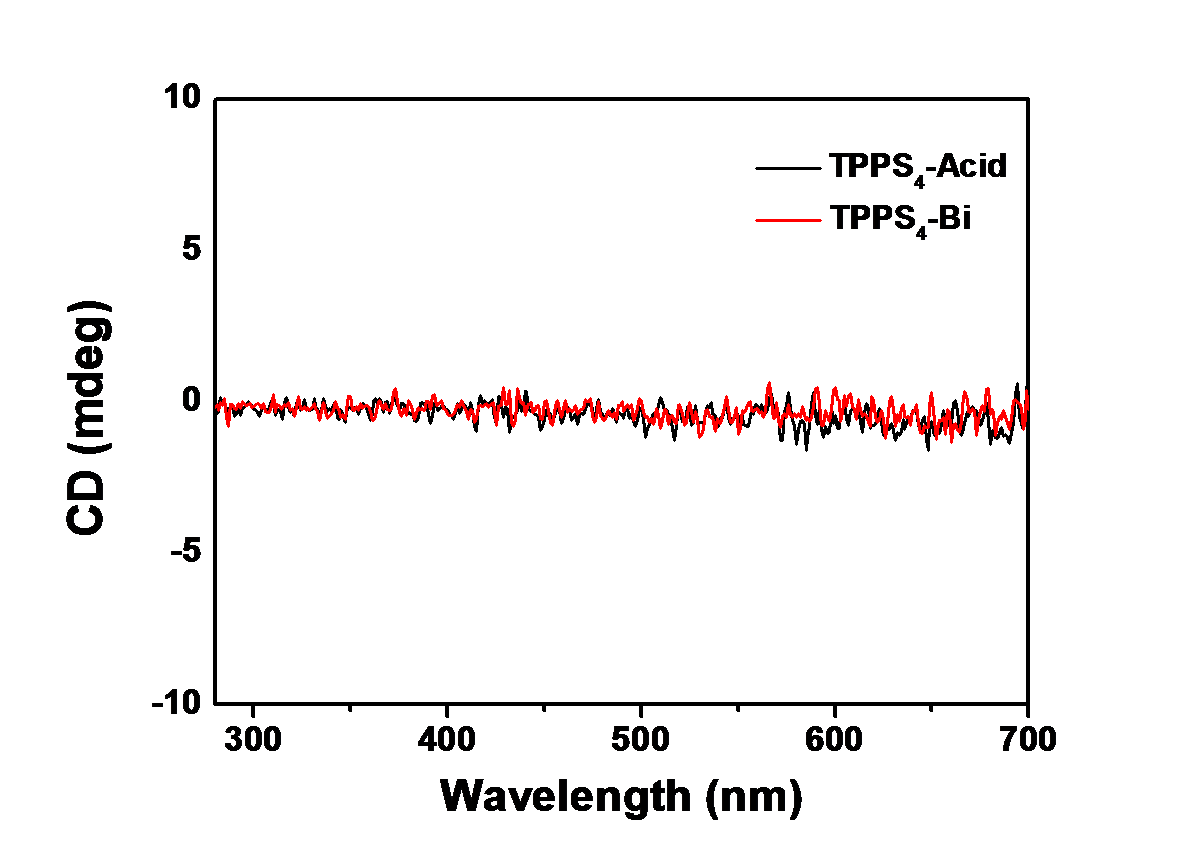


**Figure S4.** The CD spectrum of TPPS_4_-Bi and TPPS_4_ in acid aqueous solution. [TPPS_4_] = 0.025 mM, [Bi^3+^] = 0.05 mM, pH 3.6.


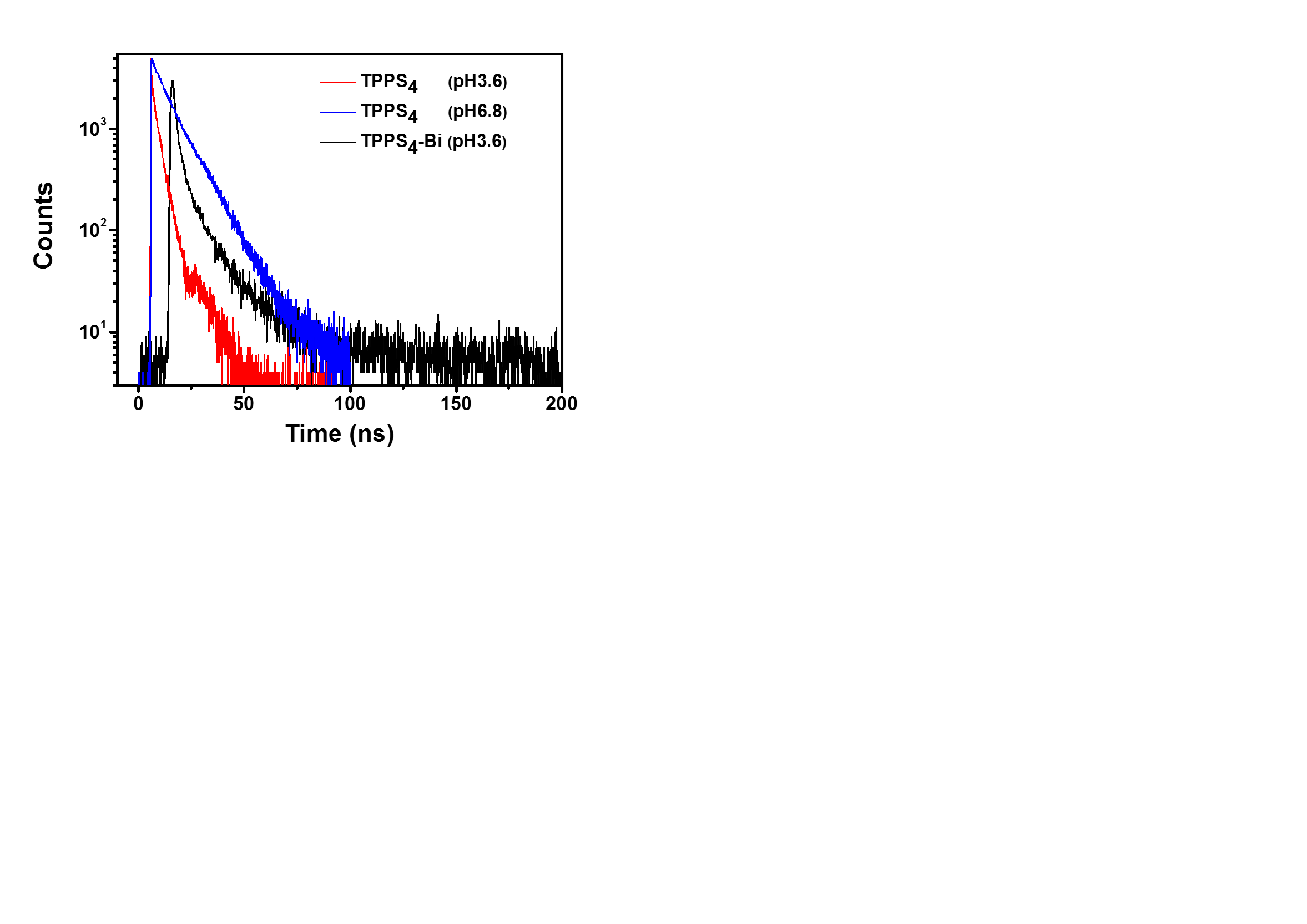


**Figure S5.** The time-resolved fluorescence of TPPS_4_ under different conditions. Excitation, 370 nm. Emission, 520 nm. Pulse length, 51.8 ps. [TPPS_4_] = 0.025 mM, [Bi^3+^] = 0.05 mM.


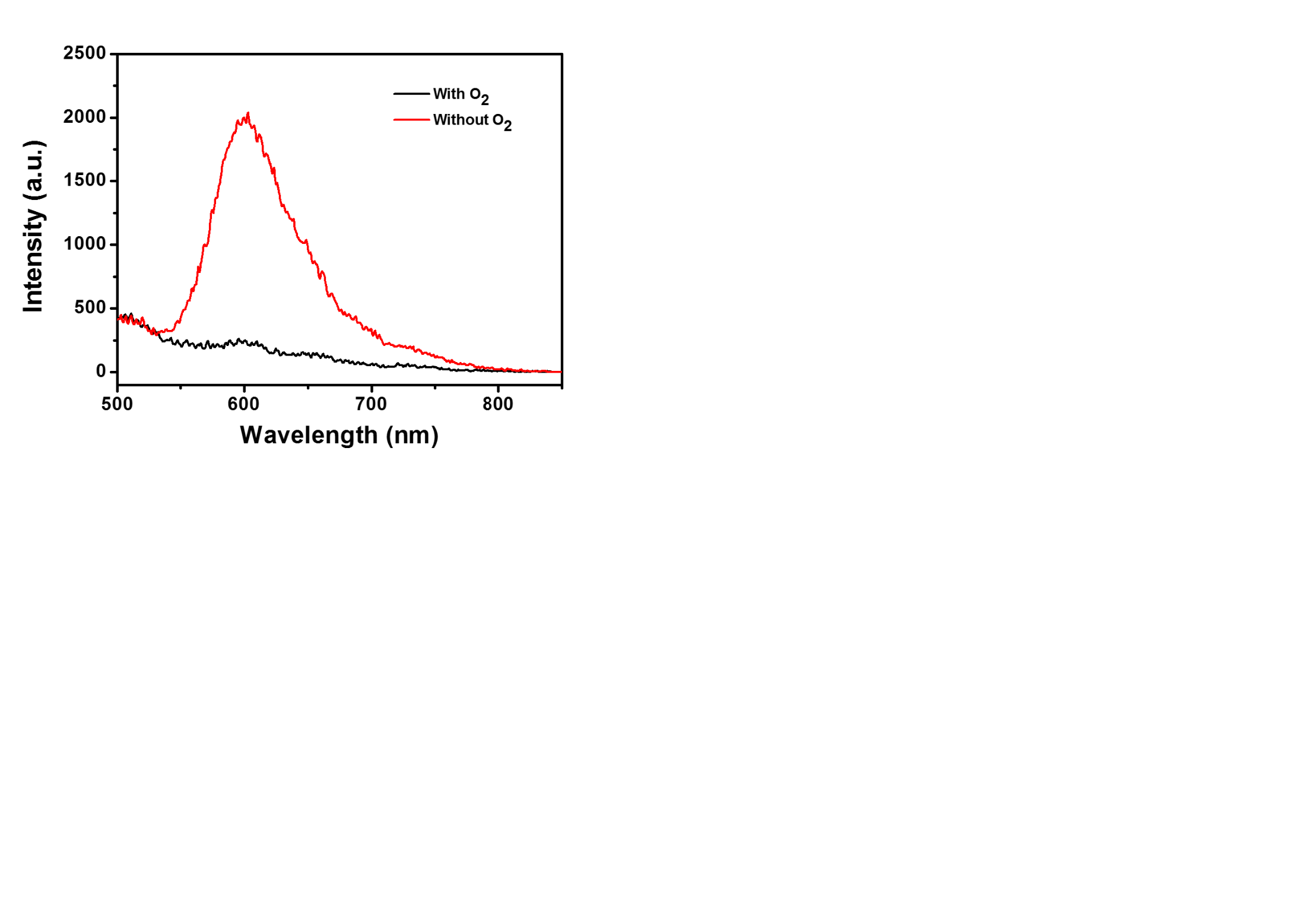


**Figure S6.** Fluorescence spectra of TPPS_4_-Bi solution with oxygen (black) or in degas environment (red). Excitation, 370 nm.


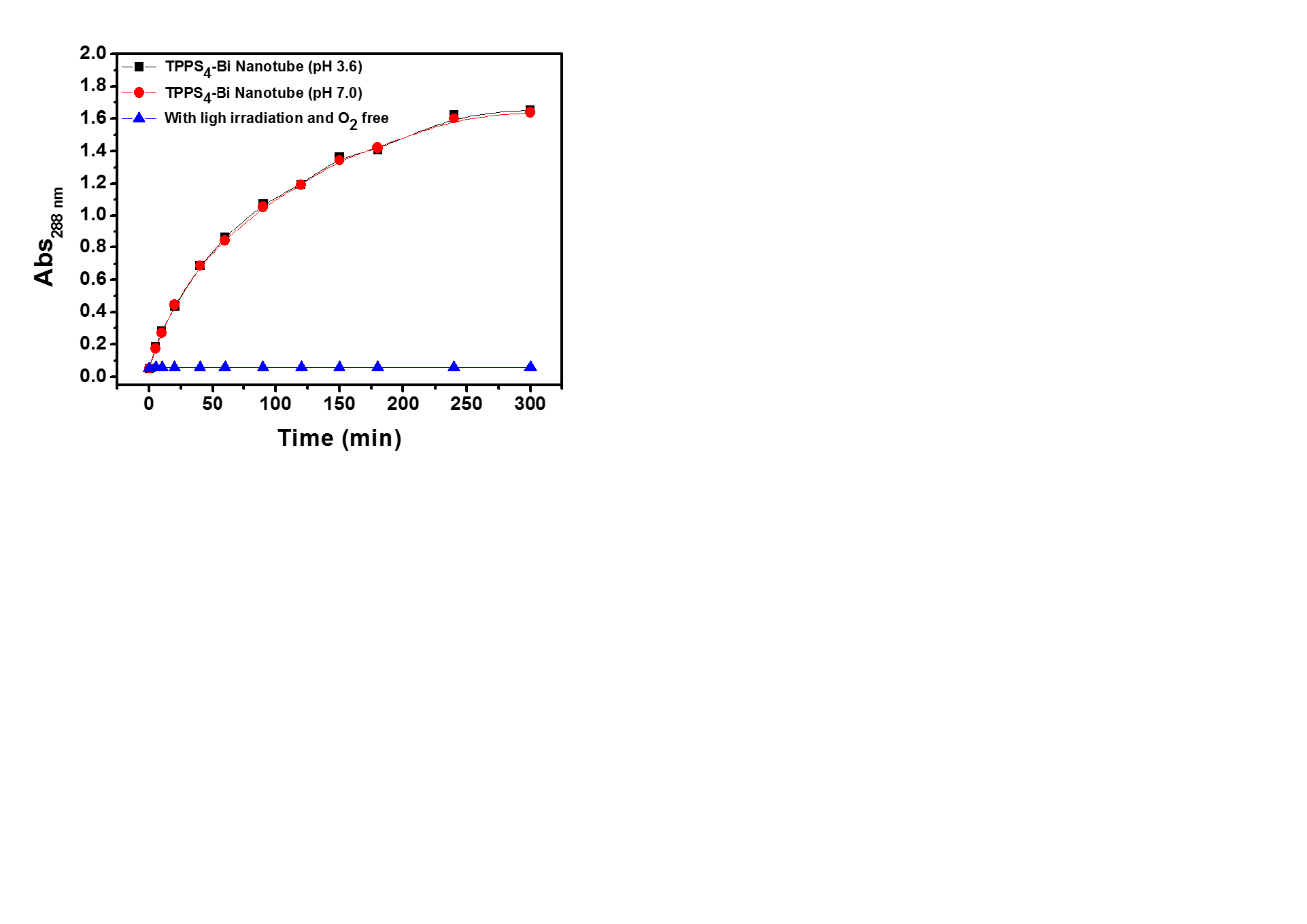


**Figure S7.** Compared ^1^O_2_ generation ability of TPPS_4_-Bi nanotube in acid solution at pH 3.6 (□) and in neutral water at pH 7.0 (○). The blank experiment of TPPS_4_-Bi nanotube in neutral water at pH 7.0 was tested under UV light and O_2_ free (△). [TPPS_4_]:[Bi^3+^] = 1:2.


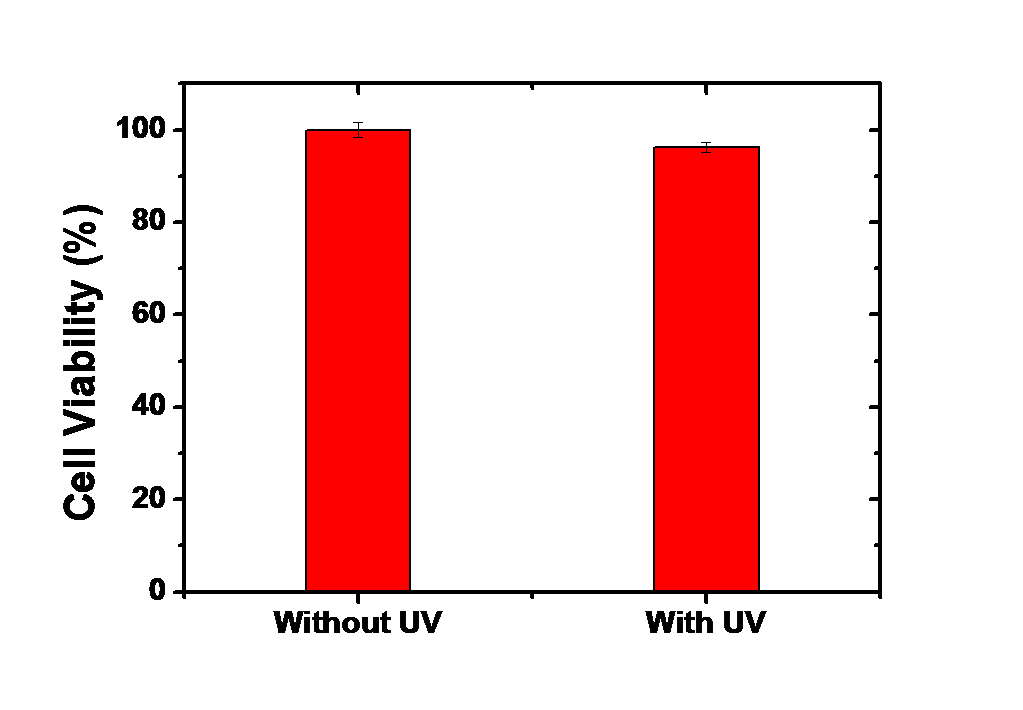


**Figure S8**. The cell viability of TPPS_4_-Bi nanotubes with and without UV irradiation. Data were presented as the mean ± standard deviation (N = 3).

**Scheme S1**. Reaction scheme of the model reaction for catalysis.


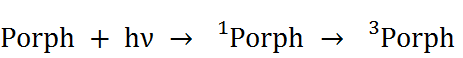


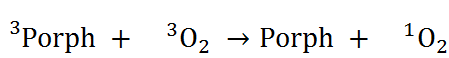


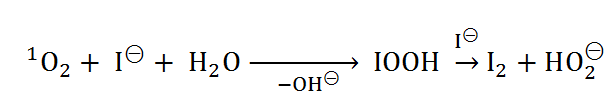


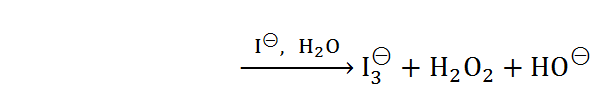


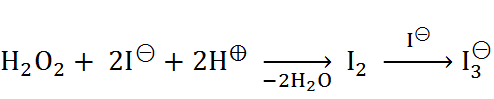

Supplement: Supplementary Information [file srep31339-s1.doc]
